# Gallbladder carcinoma: a retrospective analysis of twenty-two years experience of a single teaching hospital

**DOI:** 10.1186/1477-7800-2-6

**Published:** 2005-03-17

**Authors:** Muhammed Ashraf Memon, Suhail Anwar, M Hanif Shiwani, Breda Memon

**Affiliations:** 1 Department of Surgery, Creighton University, Omaha, Nebraska, USA; 2 Department of Surgery, Whiston Hospital, Prescot, Merseyside, L35 5DR, UK; 3 Department of Surgery, Barnsley District General Hospital, Barnsley, South Yorkshire, S75 2EP, UK; 4 Private Clinic, Astley House, Whitehall Road, Darwen, Lancashire, BB3 2LH, UK

**Keywords:** Carcinoma, Gallbladder, Human

## Abstract

**Background:**

The purpose of this study was to retrospectively evaluate our experience with gallbladder cancer since the establishment of a tumour registry in our institute.

**Methods:**

Between 1975 and 1998, 23 consecutive patients with gallbladder cancer were identified using the tumour registry database. There were 18 females (78%) and 5 (22%) males. The mean age at diagnosis was 70.6 (range 42–85) years. The diagnosis was achieved either intra-operatively or following the histological analysis of the gallbladder (n = 17), following gallbladder or liver biopsy (n = 4) or at autopsy (n = 2). Presenting symptoms included upper abdominal pain, weight loss, nausea, vomiting, fever, painless jaundice, hepatomegaly, upper abdominal mass, upper abdominal tenderness, and gastrointestinal haemorrhage.

**Results:**

Histological examination revealed 20 adenocarcinomas (87%), 2 squamous cell carcinomas (9%) and one spindle cell sarcoma (4%). At presentation, 14 (61%) gallbladder cancers were stage IV, 5 (22%) were stage III and 4 (17%) were stage II. Kaplan Meier analysis revealed a mean survival of 3.2, 7.8 and 8.2 months for stage IV, III, and II disease respectively. Out of 14 patients with stage IV disease, 8 patients received adjuvant chemotherapy and survived for 4.6 months whereas six patients who did not receive adjuvant chemotherapy survived for 1.3 months. This difference was statistically significant (p = 0.04).

**Conclusion:**

The majority of patients with gallbladder cancer presented with advanced stage disease (stage IV) which carries a dismal prognosis. Patients who received chemotherapy with stage IV disease, however, did better than those who did not, but this is probably a reflection of patient selection.

## Background

Carcinoma of the gallbladder is a rare malignancy accounting for approximately 7,100 new cases and 3,500 deaths per annum in the US. It is the most common biliary tract malignancy and the fifth most frequent gastrointestinal malignancy [1]. Its clinical presentation is non-specific and the majority of patients have advanced disease at presentation. The aim of this article is to review our experience of gallbladder carcinoma since the establishment of a tumour registry in our institute.

## Methods

Between 1975 and 1998, 23 consecutive patients with histological proven gallbladder cancer treated at St. Joseph's Hospital, Omaha, Nebraska were identified using the tumour registry database. There were 18 females (78%) and 5 (22%) males. All but one patient were Caucasian. The mean age at diagnosis was 70.6  (range 42–85) years. In 17 (74%) patients the cancer was diagnosed either intra-operatively or following the histological analysis of the gallbladder. In 4 patients, due to the extensive nature of the disease, the diagnosis was confirmed following gallbladder or liver biopsy. In the remaining two patients it was discovered at autopsy. Family history of other types of cancers was positive in 5 patients (22%), negative in 11 patients (48%) and unavailable in 7 (30%) patients. Presenting symptoms included upper abdominal pain, weight loss, nausea, vomiting, fever, painless jaundice, hepatomegaly, upper abdominal mass, upper abdominal tenderness, and gastrointestinal haemorrhage. Surgical procedures and other therapies were reviewed and their impact on survival noted. The survival of the patients discharged from the hospital was determined using Kaplan Meier analysis. P < 0.05 was considered significant. The software used was PRISM, GraphPad Software San Diego, California.

## Results

**Figure 1:**
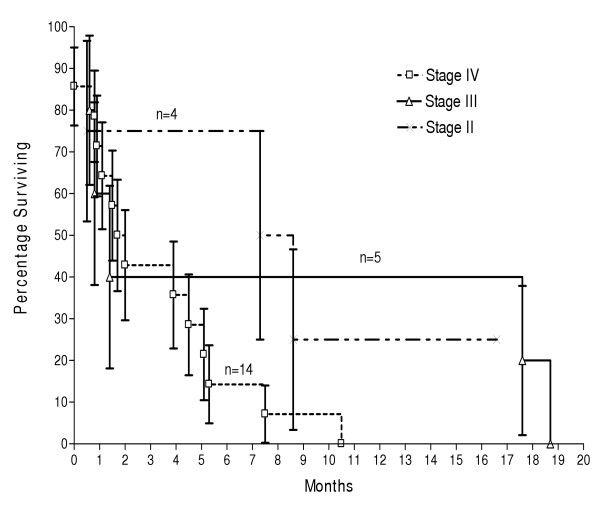
Survival of patients according to the AJCC staging classification.

Histological examination revealed 20 adenocarcinomas (87%), 2 squamous cell carcinomas (9%) and one spindle cell sarcoma (4%). At presentation, 14 (61%) gallbladder cancers were stage IV, 5 (22%) were stage III and 4 (17%) were stage II (Table 1, Table 2). Kaplan Meier analysis revealed a mean survival of 3.2, 7.8 and 8.2 months for stage IV, III, and II disease respectively (Figure 1). Only one patient was alive (16.6 months with stage II disease) at the time of analysis of this data. Out of 14 patients with stage IV disease, six patients did not receive adjuvant chemotherapy and survived for a mean period of 1.3 months. On the other hand, 8 patients who received adjuvant multi-agent chemotherapeutic treatment survived for a mean period of 4.6 months. This difference was statistically significant (p = 0.04).

**Table 1:** American Joint Commission on Cancer (AJCC) Staging

**TNM Definition**	**Tumour Location**
Tis	Carcinoma in situ
T1a	Gallbladder wall: mucosa
T1b	Gallbladder wall: muscle
T2	Perimuscular connective tissue
T3	Serosa or one organ, liver <2 cm
T4	Two or more organs, liver >2 cm
N1	Hepatoduodenal ligament nodes
N2	Other regional lymph nodes
M0	No distant metastases
M1	Distant metastases

## Discussion

DeStoll described carcinoma of the gallbladder on the bases of two autopsies in 1777 [2]. Since that time, primary carcinoma of the gallbladder has remained a uniformly fatal neoplasm. The reasons being (a) its late presentation; (b) early spread by lymphatic; haematogenous and direct route; (c) high propensity to seed the peritoneal surfaces after tumor spillage and (d) lack of effective adjuvant therapy. The majority of reports suggest that the gallbladder carcinoma is two to six times more prevalent in women and the incidence peaks in the seventh decade of life. In our series the female to male ratio was approximately 4:1 and the mean age at the diagnosis was 70.6 (range 42–85) years.

Despite advances in hepatobiliary imaging techniques [3,4], the preoperative diagnosis of gallbladder carcinoma remains a daunting task. This in part is related to the disease's non-specific presentation and its similarity to benign biliary tract disorders. In our series the majority of patients underwent ultrasound scan (USS) of the upper abdomen (on the basis of their symptoms) which revealed either the presence of gallstones with our without thick walled gallbladder or liver metastases in advanced cases. In those cases where the tumour was suspected, the findings of contrast enhanced computerised tomography (CE-CT) were not helpful either. The CE-CT findings ranged from a mass in the right lobe of the liver, dilated gallbladder, thick walled gallbladder, dilatation of intrahepatic bile ducts, abscess cavity in the right lobe of the liver and liver metastases. Other studies have similarly confirmed low sensitivity and specificity of USS and CE-CT in achieving preoperative diagnosis of carcinoma of the gallbladder [5-12]. The most common finding described for gallbladder carcinoma on both USS and CE-CT is diffuse thickening of the gallbladder. However this is also commonly reported in the inflammatory conditions of the gallbladder and therefore does not aid in the diagnosis.

In our series the majority of gallbladder carcinomas were diagnosed either intra-operatively or subsequent to histological analysis following a cholecystectomy. In the era of an laparoscopic cholecystectomy this situation poses a major dilemma as laparoscopic technique has been associated with an early and rapid dissemination of the disease both intraperitoneally and at the port site in patients with proven gallbladder carcinomas and therefore precludes potentially curative resection [13-16]. It has therefore been suggested that laparoscopic surgery should not be undertaken if radiological or clinical diagnosis of gallbladder carcinoma is suggested. Moreover, if such a diagnosis is suspected during initial laparoscopy then the procedure should be abandoned to maximize the chance of curative resection. As our experience ranged over the twenty years period, only a handful of cholecystectomies were performed laparoscopically and in all these patients the diagnosis was revealed only after the histological analysis of the specimen.

The overall outcome of this disease is dismal with the 5-year survival rate being less than 5% with a median survival of 5 to 8 months. Piehler and Circhlow [17] reviewed 5836 patients with carcinoma of the gallbladder from 1960 to 1978 and found an overall 5-year survival of 4.1% and one year survival of 11.8%. Only 25% were resected for cure and of these only 16.5% survived 5 years. However this figure dropped to 2.9% if the surgeon identified a tumour at the time of exploration. The best survival was achieved in patients whose cholecystectomy specimens were found to have incidental tumour. Even then only 14.9% survived 5-year. Similarly Cubertafond et al [18] reported a median survival of 3 months, a 5-year survival of 5% and a 1-year survival of 14% amongst 724 carcinomas of the gallbladder. They observed no differences among the different surgical procedures adopted and concluded that no progress had been made in the treatment of gallbladder carcinoma. This clearly demonstrates the minimal impact that surgical treatment has had on this disease. However two recent reviews from Japan have contradicted all these previous reports. Ogura et al [19] have reported an impressive 50.7% 5 year survival for 984 patients undergoing radical resection compared to only 6.2% for 702 patients undergoing conservative management. Similar Todoroki et al [20] have also shown that radical resection for gallbladder carcinoma improves the prognosis even for stage IV disease, provided that complete gross tumour resection is combined with radiotherapy. These results suggest the possible role of surgery ± adjuvant therapy in changing the natural history of gallbladder carcinoma. In the current series none of the patients underwent any extensive surgical resection either because of incidental findings of gallbladder carcinoma following a cholecystectomy or the tumour was too extensive at the time of laparotomy. The longest survival recorded was 18.7 months with a median survival of 5.1 months. Subset analysis revealed that patients with well differentiated adenocarcinoma had the best outcome although none of them survived even two years.

The stage at which the disease presents certainly has a direct impact on the survival of the patient (Table 1, 2). A number of authors have reported between 71% to 100% 5 year actuarial survival for stage I carcinoma both following a simple and extended cholecystectomy [21,22], between 22% to 100% for stage II carcinoma only following an extended cholecystectomy [23,24], between 8% to 63% for stage III carcinoma [25,26] and between 8% to 25% for stage IV carcinoma following an extended resection ± adjuvant therapy [19,26]. We have not been able to demonstrate such impressive survival figures in our patients. The survival of our patients certainly was better in the lower stage disease compared to the advanced stage yet no patient even made it to 2 years (Figure 1). This may be due to the fact that an extended cholecystectomy was not performed in any of our patients. This validates the point raised by Ogura et al [19] in their review that extended therapy improves long term survival.

**Table 2:** Staging of gallbladder carcinoma

**Stage**	**TNM**	**Modified Nevin**
0	Tis N0 M0	In situ carcinoma
I	T1 N0 M0	In situ carcinoma
II	T2 N0 M0	Mucosal or muscular invasion
III	T1 N1 M0 T2 N1 M0 T3 N0 M0 T3 N1 M0	Transmural direct liver invasion
IV A	T4 N0 M0 T4 N1 M0	Lymph node metastasis
IV B	Any T N2 M0 Any T Any N M1	Lymph node metastasis ± distant metastasis

Adjuvant chemotherapy and/or radiotherapy for patients with gallbladder cancer has not altered the dismal prognosis, but may marginally improve survival. Chao et al [27] reported no survival benefit between the two groups of patients, one receiving and other denied adjuvant chemotherapy and/or radiotherapy. On the other hand Oswalt and Cruz [28] and Morrow et al [29] have shown an improved median survival amongst the cohort of patients receiving chemotherapy and/or radiotherapy following their surgery. Similarly Makela and Kairaluoma [30] have demonstrated that superselective intra-arterial chemotherapy with mitomycin for gallbladder cancer had a 48% response rate and the responder had a significantly better survival (34 months) compared to the non-responders (8 months). However this type of therapy was only effective in those patients whose tumours were confined to gallbladder wall. Similarly in our study, patients who received adjuvant multi-agent chemotherapeutic treatment for stage IV disease had a significantly longer mean survival period compared to the ones who did not receive such treatment. This may simply reflect patient selection bias and therefore it is impossible to credit minimal improvements in survival in ours and other series to chemotherapy alone without a large randomised trial. An important point to make is that none of the patients who received chemotherapy in our series survived beyond two years – indeed a disappointing outcome.

## Conclusion

Gallbladder carcinoma not only presents a diagnostic dilemma but also poses a difficult treatment option in the era of laparoscopic cholecystectomy. The majority of patients with gallbladder cancer present with advanced stage disease (stage IV) which carries a dismal prognosis. Chemotherapy seems to have some survival benefit in stage IV disease, but no randomized controlled trials exit to define its role in the adjuvant setting. The prognosis of this disease is dismal and even a 2 year survival seems to be the exception rather than the rule.

## Authors' contributions

MAM was responsible for acquisition, analysis and interpretation of data; MAM, SA, MHS and BM have been involved in drafting the manuscript and revising it critically for important intellectual content and have given final approval of the version to be published. All authors have participated sufficiently in the work to take public responsibility for its content.
